# A Night Out with the Nerds

**DOI:** 10.1371/journal.pbio.0030325

**Published:** 2005-09-13

**Authors:** Sandra Knapp, James Mallet

## Abstract

Simon Singh and Richard Wiseman draw on examples from physics to psychology, to explore the extraordinary in the ordinary in their innovative new play *Theatre of Science*

Add performance to science, and the equation results in *Theatre of Science*. Part scientific lecture, part magic show, and part music and dance, *Theatre of Science* is an innovative collaboration between Simon Singh and Richard Wiseman. The cosy atmosphere of the Soho Theatre, stuffed with a lively crowd, which appeared swelled by a smattering of family and friends, makes the show a personal interchange between performers and audience. Several in the audience must have been scientists, judging by appearances: one enthusiastic member of the audience was a dead ringer for the recently outgoing president of the Royal Society, only taller and more etiolated. Every show will be different, depending on the audience's reaction. Wiseman, in a warm-up session, explicitly “titrates” the audience for its sense of humour, before embarking on the rest of the show. Here is an example: “We were hoping that our contortionist Delia would be here tonight, but, I promise you, this is not a joke, she called in a few moments ago to say she was tied up in traffic”. With the audience ready, Singh demonstrates “the scientific method” by proving mathematically that Teletubbies = evil. As a hint, the proof involves the well-known law, “Money is the root of all evil”.

Using amusing and wacky examples from physics and psychology, Singh and Wiseman explore the extraordinary in the ordinary. Ground-breaking science often emerges from massive pieces of equipment, such as mile-long linear accelerators or radio telescopes, but science—much of it still unexplored—pervades the seemingly ordinary, everyday world as well. For example, we are now convinced of profound “holes in our understanding” of the physics of balloons. (How *did* he put that knitting needle through, and then withdraw it again, without the balloon popping?)

Singh is a well-known broadcaster, popular science writer, and a successful scientist who obtained his PhD in physics at Cambridge University and the European Organization for Nuclear Research (CERN) (see http://www.simonsingh.net). Wiseman is a magician (a failed magician, he says, as he deliberately drops a card he has palmed). Instead, he is now the world's only Professor of Public Understanding of Psychology, at the University of Hertfordshire (see http://www.richardwiseman.net). Wiseman's forte is optical illusion, the magician's stock-in-trade, but most impressive is his exploration of quirks of our perception of ordinary things. “Psychologists today earnestly debate if anything we see is real at all”, he says. If you think you are a great observer—and as scientists we are generally proud of our powers of objective observation—just relax and participate in *Theatre of Science*. You'll be amazed how easily you can be tricked by Wiseman's ruses.

Mixed in with Singh's three-minute explanation of the Big Bang (more or less, if you exclude his five-minute appendix), we see a clear demonstration of the inevitability of coincidence. In *The Bible Code*, Michael Drosnin argues that the Bible contains many prophecies of today's events; however, the *Theatre of Science* demonstrates that Herman Melville's *Moby Dick* has even more startlingly correct predictions…about Princess Diana's death, for instance. In another piece, an alarmingly glowing orange and sparking gherkin is followed by beautiful, weird sounds from theramin player Sarah Angliss and her assistant. The theramin is an electronic musical instrument consisting of a rod and a hoop, both attached to tuned circuits that are connected to speakers. The theramin is played with hand and body movements, which affect volume and pitch; it is as much dance as it is music, and the sound is ethereal.

Contortionists make us wince—but how do they do that with a body that looks just like ours? Is it their bones, their ligaments, or something quite different? Inserting contortionist Delia Du Sol into a whole-body magnetic resonance imaging scanner provides the answer—the images tell an amazing story. (Guess! We're not giving it away.) Then Du Sol's performance—accompanied by theramin music and overlaid with recordings of her talking about how she felt while performing, in particular how she hoped that people didn't perceive her as a freak—opened up a new dimension. The contrast between her matter-of-fact speech and the tension in the audience was unnerving.

Although the show doesn't end with a thunderous bang, it does have real lightning—every bit as good. This final experiment (or do we mean skit?) requires two six-foot-high, out-of-phase, coupled Tesla coils producing a combined total of a million volts, we are told. Initially, in spite of the big numbers, we aren't impressed by the voltage (don't combs generate similar voltages in dry hair?), but when we see and hear the arcs jumping six feet and smell the burnt air (thought to be caused by nitrogen/oxygen compounds forming in the plasma created by the arcs), we realize there are quite a few amps as well as volts. Audience tension is slowly and carefully building. The electrifying finale is no set-up job, and there is real potential for danger. Those wearing pacemakers are advised to leave the room to avoid certain death. This is supreme showmanship, part circus, part laboratory, and part drama. Do we, the audience, viscerally believe in scientific theory?

We vote on whether Singh or Wiseman will occupy the Faraday cage, also known as the “coffin of terror”, being inserted between the “coils of death”. We see and hear lightning strike the humanoid form (which looks like it was constructed to house the son of Frankenstein) now containing Wiseman. After the discharges, the sounds die away, and there follows a long period of silence and immobility. Singh then moves to slowly and deliberately open the Faraday cage – the tension mounts. Suddenly, Wiseman leaps out of the “coffin of terror”, saying “the physics was right, thank you!”

The equation works, and we can be proud to be nerds. It is a blend of science, humour, and performance that might not have worked at all, but it most emphatically did, and we recommend it as a great night out for scientists and nonscientists alike. We hope for a return engagement soon!
